# Exploring the role of intergenerational programs in promoting active aging: views and experiences of older adults in Sichuan Province

**DOI:** 10.3389/fpsyg.2026.1782592

**Published:** 2026-04-14

**Authors:** Nengyan Yuan, Chatchai Rakthin, Nur Fauziyah, Sri Suryanti

**Affiliations:** 1 Department of Science Innovation and Culture, Institute of Science Innovation and Culture, Rajamangala University of Technology Krungthep, Bangkok, Thailand; 2 Institute of Science Innovation and Culture, Rajamangala University of Technology Krungthep, Bangkok, Thailand; 3 Mathematics Education Department, Faculty of Mathematics and Natural Sciences, Universitas Negeri Surabaya, Surabaya, Indonesia

**Keywords:** active aging, community involvement, health index, intergeneration program, self-development

## Abstract

Although existing research highlights the benefits of intergenerational interactions in enhancing social support and reducing isolation, limited studies have examined how such programs specifically contribute to the health and well-being of older adults. The aim of this research is to investigate how older adults perceive their participation in intergenerational programs in terms of physical, mental, and social engagement, and how these programs support their overall active aging process. Using a mixed-methods design, the study collected data from 70 participants of older adults engaged in intergenerational, through surveys and semi-structured interviews. Data analysis was conducted using thematic analysis for qualitative data and descriptive statistics for quantitative data. The findings reveal that older adults involved in intergenerational programs report increased social engagement, improved mental health, and greater feelings of autonomy. These participants also expressed a heightened sense of community and intergenerational solidarity. The study implies that intergenerational programs are a valuable strategy for enhancing active aging and should be integrated into public health policies aimed at supporting aging populations.

## Introduction

**Intergenerational programs** to promote active aging are initiatives designed to bring together different generations, particularly older adults and younger individuals, in collaborative activities that foster mutual learning, understanding, and support (Murray et al., 2025; Pinazo-Hernandis and Carrascosa, 2025). These programs can take many forms, from mentorship opportunities to shared community projects, all aimed at improving social connections, enhancing life skills, and encouraging active participation in society (Leong et al., 2022; Petersen, 2022). Through these programs, older adults can experience greater engagement, social inclusion, and empowerment, which are essential components of active aging (Leong et al., 2022; Teater, 2016).

Intergenerational programs are often recognized as tools for promoting active aging among older adults. They offer more than just opportunities for challenging stereotypes and fostering better intergenerational understanding (Gutiérrez-Domingo, 2024). These programs can play a crucial role in promoting health, self-development, and community building. According to the World Health Organization (WHO) (2002), intergenerational activities are seen as a means of enhancing the quality of life for older individuals by increasing social interactions, providing cognitive stimulation, and helping them stay physically and mentally active (Khalil, 2024). However, there is limited research examining how such programs directly contribute to active aging from the perspective of older adults themselves (Hwang et al., 2024; Rabinowitz et al., 2022).

This study aims to address this gap by exploring the extent to which older adults in Sichuan Province China perceive their involvement in intergenerational programs as beneficial for their overall well-being. Using a survey methodology with both open- and closed-ended questions, the research examines how participation in these programs contributes to older adults’ physical and mental health, personal development, and sense of community. The findings shed light on what older participants feel, think, and learn through their involvement, providing valuable insights into how intergenerational activities can be tailored to maximize benefits for active aging.

## Literature review

### The conceptualization of older adults’ active aging

Active aging is a comprehensive framework that aims to optimize opportunities for health, participation, and security to enhance the quality of life as individuals age. Developed by the World Health Organization (WHO) in 2002, this concept extends beyond physical activity to include active involvement in social, economic, cultural, and civic affairs. It is underpinned by six determinants: economic, social, health and social services, behavioral, personal, and physical. Each of these determinants interacts with gender and cultural factors, making the concept contextually significant. Of particular importance, the social determinant emphasizes social support as crucial to active aging, where a loss of social activity or support can lead to isolation, loneliness, and diminished well-being. This intergenerational solidarity—interaction between older and younger generations—is highlighted as a critical aspect of active aging, facilitating mutual aid and emotional support, which directly contribute to the overall quality of life for older adults (de Paula Sieverding et al., 2024; Hwang et al., 2024).

The practical application of active aging can be examined by understanding older adults’ own perceptions of the concept. Research by Bowling (2008) and Stenner et al. (2011) provides insight into how older adults define active aging. Bowling’s study found that physical health and leisure activities were central to participants’ understanding of active aging, followed by mental functioning and social relationships. Similarly, Stenner et al. (2011) noted that older adults frequently linked active aging to physical movement, mental activity, and social engagement, but also highlighted autonomy and self-sufficiency as essential components. This reveals that active aging is not merely about physical activity but about maintaining independence, making choices, and engaging in activities that foster a sense of control over one’s life. It underscores the importance of older adults viewing themselves as active agents in their aging process, rather than passive recipients of care.

Additionally, the research indicates that active aging is a dynamic concept shaped by individual choice and response to challenges. Stenner et al. (2011) emphasized that older adults define active aging as the ability to engage in meaningful activities while maintaining the autonomy to choose how to live, despite challenges such as functional disabilities or societal discrimination. These challenges are viewed not as constraints but as opportunities for older adults to exercise agency in determining how they respond. The study suggests that the subjective meaning and interest of activities are closely tied to an individual’s sense of autonomy and the ability to remain actively involved in life (Saito et al., 2020). Thus, active aging encompasses a balance of physical, mental, and social activity, autonomy in decision-making, and the resilience to navigate challenges without being defined by them. This holistic approach to aging integrates both individual empowerment and the need for external social and intergenerational support, essential for promoting a fulfilling and engaged life in later years.

### Research on intergenerational programs to support active aging

Research on intergenerational programs has highlighted their effectiveness in addressing age-related stereotypes, promoting attitudinal shifts, and offering various benefits to older adults. These programs have been recognized as a key element in active aging policies, which emphasize the importance of fostering social support for the elderly. Participation in such programs enables older individuals to remain socially engaged, which is widely considered essential for maintaining an active and fulfilling life.

Moreover, older adults themselves frequently report that being socially connected and engaged in diverse activities is a critical component of active aging, making intergenerational programs a valuable strategy to enhance their quality of life. For example, a qualitative study conducted in Singapore found that older adults participating in intergenerational programs at a senior day care center reported increased feelings of social connection, uplifting experiences with younger participants, and greater opportunities for meaningful activity and engagement in daily life (Leong et al., 2022). Complementing this, a systematic review of intergenerational exchange programs highlighted consistent evidence across multiple countries that such initiatives improve psychosocial and cognitive outcomes among older adults, including enhanced social cohesion, greater self-awareness, and improved physical functioning, though methodological variability remains a challenge (Furuto, 2019; Murray et al., 2025; Pinazo-Hernandis and Carrascosa, 2025). These findings align with broader evidence suggesting that intentional intergenerational engagement — whether through formal programs or shared community activities — supports facets of active aging by facilitating ongoing social participation and reducing isolation.

Meta-analytical research further reinforces the role of intergenerational programs in promoting aspects of active aging such as quality of life and mental health. A meta-analytic review of non-familial intergenerational programs reported that older participants experienced statistically significant improvements in quality of life and reductions in depressive symptoms, alongside increases in generativity — the sense of purpose derived from contributing to younger generations (Petersen, 2022). Qualitative studies conducted in diverse settings, including residential care facilities, also found that intergenerational interactions with children or adolescents were associated with positive affective experiences, increased sense of belonging, and supportive emotional outcomes for older adults, particularly in reducing loneliness and enhancing mood (Earl and Marais, 2023). Collectively, these multinational findings suggest that intergenerational engagement is more than a stereotype-challenging tool; when structured and contextually adapted, it contributes meaningfully to the psychological and social dimensions of active aging.

Finally, narrative reviews and mini-reviews from Europe and Spain underscore intergenerational programs as strategic supports for active aging policies by strengthening community ties and fostering reciprocal learning between generations. For instance, a narrative review focusing on intergenerational projects in Spain concluded that initiatives such as “Adopt a Grandparent” help alleviate loneliness, build diverse social networks, and enhance health and emotional well-being, supporting the participation and integration pillars central to the World Health Organization’s active aging framework (Gutiérrez-Domingo, 2024). These psychosocial benefits are substantiated across different cultural contexts, demonstrating that intergenerational programs can act as effective mechanisms for community cohesion and multisectoral engagement, ultimately contributing to healthier aging trajectories when older adults are viewed as active, valued participants in shared social life (Murray et al., 2025; Pinazo-Hernandis and Carrascosa, 2025).

This exploratory study aims to delve deeper into how intergenerational programs can directly impact the health, well-being, and personal development of older adults. The study also seeks to provide insights into how intergenerational programs align with the principles of active aging, specifically from the perspective of older participants themselves, by posing the following research questions:

1. What are the thoughts, emotions, and learnings that older adults experience through their participation in these programs?
2. How do older adults perceive their involvement in intergenerational programs as contributing to their health, self-development, and community building?

## Methods

### Research design

The study employs a mixed-methods design following Creswell and Clark (2017) combining both quantitative surveys and qualitative interviews to explore how participation in an intergenerational program enhances the perceived health and well-being of older adults. A survey is used to capture broad, generalizable insights from a large group of participants, offering quantitative data that reveals patterns and trends related to the program’s impact. Additionally, in-depth interviews are conducted to gain richer, more nuanced perspectives from a smaller subset of participants, providing qualitative insights into their personal experiences and reflections. This mixed-methods approach is particularly valuable as it allows for the triangulation of data, enhancing the validity of the findings by combining numerical data with individual narratives to offer a more comprehensive understanding of the program’s effects on older adults’ health and well-being.

### Participants

The survey was distributed to a total of 80 participants; however, 70 participants completed and submitted their responses, and only these responses were included in the final analysis. These participants were recruited from multiple community-based intergenerational programs across Sichuan Province and consisted of two groups: 70 older adults who completed the survey and 7 older adults who participated in in-depth interviews. All participants were aged between 60 and 78 years, reflecting the program’s target population of community-dwelling older adults. In the survey group (*n* = 70), 42 participants were female (60%) and 28 were male (40%), while the interview subgroup (*n* = 7) comprised 4 females and 3 males. This gender distribution reflects the typical demographic composition of community-based aging programs in the region, where female participation tends to be slightly higher.

Participants were selected from various intergenerational initiatives implemented in urban and semi-urban areas of Sichuan, all of which aimed to promote active aging, social engagement, and community integration. For the quantitative phase, purposive sampling was employed to ensure that respondents had participated in the program for a minimum of 3 months, thereby allowing sufficient exposure to assess perceived impacts on health and well-being. Recruiting participants from multiple community centers enhanced diversity in terms of age range, gender, educational background, and socioeconomic status. The sample size of 70 was considered adequate to provide statistically meaningful insights into overall trends and perceived program outcomes, while the smaller qualitative subsample enabled deeper exploration of individual experiences within the same demographic framework.

In contrast, the interview participants were selected using a more targeted purposive sampling approach, focusing on individuals who had been engaged in the program for an extended duration and who were willing to provide detailed, reflective insights into their experiences. The seven interviewed participants (4 females and 3 males) were selected from a standardized program delivered across multiple community centers in Sichuan, ensuring representation of diverse personal backgrounds and varying levels of engagement. These participants were selected based on their willingness to engage in more in-depth discussions and their ability to reflect on the nuances of their participation. This selection process allowed the research team to gather rich, qualitative data that complemented the survey findings, providing a deeper understanding of how the program impacted the older adults’ sense of health, development, and community connection.

### The detailed description of intergenerational activity program

To enhance research transparency and replicability, this project adopted a structured operational model designed to promote active aging through sustained intergenerational engagement. The primary objectives were: (1) to strengthen older adults’ social participation and sense of community contribution; (2) to facilitate reciprocal learning and mutual understanding between younger and older generations; and (3) to reduce social isolation by embedding older adults within routine community-based collaborative activities. The program was grounded in a participatory and strengths-based framework, positioning older adults not as passive recipients but as knowledge holders, mentors, and cultural transmitters. Clear role delineation, standardized session plans, and documented facilitation guidelines were developed to ensure consistency across implementation cycles.

In terms of activity formats and practical content, the project combined thematic workshops, skill-sharing sessions, dialogue circles, and collaborative community projects. Workshops included digital literacy training, local history storytelling, handicraft production, and health-promotion education, each designed to encourage two-way interaction rather than one-directional instruction. Dialogue circles were facilitated using semi-structured guiding questions to promote reflection on community values, generational identity, and shared social challenges. Collaborative projects—such as neighborhood improvement initiatives or cultural exhibitions—required mixed-age teams to plan and execute tangible outputs, thereby reinforcing cooperative problem-solving and shared responsibility. All activities followed a standardized session structure: ice-breaking (10–15 min), thematic engagement (40–60 min), collaborative task (30–45 min), and reflective discussion (15–20 min).

The program was implemented on a biweekly basis, with each session lasting approximately 2–3 h. Participation was voluntary and recruitment was conducted through local community centers and neighborhood committees, ensuring accessibility for older adults with varying educational and socio-economic backgrounds. Younger participants were recruited through schools and youth organizations within the same locality to strengthen place-based community ties. A registration and attendance tracking system was maintained to monitor engagement levels and retention rates. Each implementation cycle lasted 6 months, comprising 10–12 structured sessions, followed by a community showcase event to consolidate outcomes.

Organizationally, the project was jointly coordinated by community service centers, local aging associations, and educational institutions. A designated project coordinator oversaw logistics and fidelity to the operational framework, while trained facilitators—typically social workers or educators—guided session delivery. Regular facilitator briefings and reflective meetings were conducted to ensure consistency and allow minor contextual adaptations without compromising core program components. This clearly defined structure, standardized content modules, and documented implementation schedule provide a replicable template for similar intergenerational initiatives in comparable community settings.

### Research instruments

#### Questionnaire design

The questionnaire was systematically developed based on the study’s theoretical framework to ensure conceptual alignment with the core constructs under investigation. Specifically, the instrument operationalized three dimensions derived from the Index of Arts as Self-Health Enhancer, Index of Arts as Self-Developing Activities, and Index of Arts as Community Builders (Michaols and Kahlke, 2010; Teater, 2016). These indices provided the conceptual foundation for measuring perceived improvements in physical and psychological well-being, personal growth and self-efficacy, and community engagement resulting from participation in the intergenerational program.

To ensure content validity, the initial pool of items was evaluated by three independent experts in arts-based community interventions, gerontology and public health, and social science research methodology. The experts assessed item clarity, construct relevance, and representativeness. Their feedback led to minor revisions in wording, item sequencing, and construct coverage to improve conceptual precision and contextual suitability. Prior to full-scale administration, the revised instrument was piloted with 25 older adults who shared similar demographic and socio-cultural characteristics with the targeted sample but were not included in the main study. The pilot test assessed clarity, response consistency, and preliminary reliability, resulting in further refinement of ambiguous items.

The final questionnaire consisted of 16 Likert-type items rated on a five-point scale (1 = strongly disagree to 5 = strongly agree). The targeted sample comprised older adults (aged 60 years and above) who had participated in the intergenerational program for at least 3 months. Internal consistency reliability was evaluated using Cronbach’s alpha coefficients. The overall scale demonstrated strong reliability (*α* = 0.91), while the subscales also showed satisfactory to excellent internal consistency: Self-Health Enhancement (α = 0.88), Self-Development (α = 0.86), and Community Building (α = 0.89). These values indicate that the instrument possesses adequate psychometric robustness for assessing perceived health and well-being outcomes in this population.

### Interview protocols

The interview protocol for this study was designed to complement the questionnaire by providing deeper insights into participants’ experiences and perspectives regarding the intergenerational program. The interview questions were developed based on the theoretical framework, ensuring they addressed key dimensions of the program’s impact on older adults’ health, development, and community engagement (Michaols and Kahlke, 2010; Teater, 2016). The protocol was structured to elicit detailed, qualitative responses that would allow for a more comprehensive understanding of how the program influences participants on a personal and social level.

To ensure the validity and relevance of the interview items, the protocol was reviewed by three experts in the fields of arts, health, and social sciences. Their feedback guided the refinement of the questions, ensuring they were clear, unbiased, and aligned with the study’s theoretical constructs. The final interview protocol included open-ended questions designed to encourage participants to reflect on their personal experiences. Examples of interview items include: “Can you describe how your participation in the program has affected your physical health?” and “In what ways has the program helped you feel more connected to your community?” These questions were intended to capture both the personal and communal aspects of participation, allowing participants to provide rich, detailed responses that reflect the nuanced impact of the program on their well-being and social engagement.

### Data collection and analysis

The data collection process for this study was conducted in two phases: survey data collection and interview data collection. For the survey, participants were asked to complete a questionnaire that included 16 Likert-style items derived from the Index of Arts as Self-Health Enhancer, Index of Arts as Self-Developing Activities, and Index of Arts as Community Builders (Michaols and Kahlke, 2010). The questionnaire was administered in person and online to 70 participants across various community centers in Sichuan Province.

Participants were informed about the study’s purpose and their voluntary participation before filling out the questionnaire. The survey aimed to assess their perceptions of the impact of intergenerational programs on their health, development, and community engagement. For the interviews, seven participants were selected based on their prolonged engagement in the intergenerational program and willingness to provide detailed insights into their personal experiences. Semi-structured interviews were conducted in person and were recorded with participants’ consent. These interviews aimed to explore participants’ in-depth perspectives on how the program affected their physical health, mental well-being, and sense of community connection. The interviews were designed to allow participants to share their experiences openly and provide detailed accounts of their interactions with the program.

To analyze the survey data, descriptive statistics were used to summarize participants’ responses to the 16 items on the three Indexes. The frequencies and percentages of participants who responded with “strongly agree,” “agree,” “undecided,” “disagree,” and “strongly disagree” were calculated for each item. For one item of the questionnaire, participants were asked to rate their experience as “poor,” “fair,” “good,” or “excellent,” and these responses were also analyzed for frequencies and percentages. Additionally, the mean and standard deviation for each item on the Indexes were calculated to assess the overall trends in participants’ responses. Each of the three Indexes was analyzed by summing the scores across the items and calculating the overall mean. The Indexes have different potential total scores: the Index of Arts as Self-Developing Activities and the Index of Arts as Self-Health Enhancers range from 6 to 30, and the Index of Arts as Community Builders ranges from 4 to 20. Higher mean scores indicate more positive results in the participants’ perceptions. Bivariate analyses were conducted to determine whether there were significant correlations between older adults’ age, time spent in the program, and type of contact with their mean scores on each Index.

For the interview data, a thematic analysis approach (Guest et al., 2012) was used to identify common themes across participants’ responses. The analytic process followed a rigorous multi-stage approach: (1) familiarisation with the data through repeated reading of transcripts, (2) initial open coding to capture meaningful units related to participants’ experiences, (3) grouping of similar codes into sub-themes, and (4) abstraction of these sub-themes into three overarching themes—HI, SDI, and CBI—based on conceptual similarity and alignment with the study objectives. First, the Health Index (HI) captures insights related to participants’ physical, mental, and emotional well-being as influenced by the intergenerational program. Second, the Self-Development Index (SDI) reflects aspects of personal growth, including skills enhancement, self-efficacy, and learning experiences gained through participation. Third, the Community Building Index (CBI) encompasses findings on social interaction, sense of belonging, and the strengthening of intergenerational relationships within the community. This thematic structuring enables a more focused interpretation of how the program contributes across health, individual development, and community engagement dimensions.

Ethical clearance was obtained from the relevant institutional review board to ensure the study adhered to ethical standards. Participants were informed about the study’s objectives, and their informed consent was obtained prior to participation in both the survey and interview phases. The confidentiality of participants’ responses was maintained, and all data were anonymized to protect their identities. Triangulation was employed to enhance the validity of the study’s findings. By collecting both quantitative survey data and qualitative interview data, the study was able to cross-check and validate findings from different data sources. This approach helped to provide a more comprehensive understanding of the intergenerational program’s impact from both a broad and in-depth perspective.

### Findings

Research Question (RQ1): What are the thoughts, emotions, and learnings that older adults experience through their participation in these programs?

Perceived Contribution to Self-Development, Health, and Community Building. Table 1 describes the responses of participants toward the three Indexes in the forms of the frequencies and percentages. The results of analysis in indicated that 0% of the older adults participants reported strongly disagree to all of the 16 questionnaire items. In regard to the dimension of Arts as Self-Development Index, the majority of participants agreed or strongly agreed that their active participation in the “intergeneration event” contributed to their confidence growth (84.3%), facilitated them to learn about their life (71.4%), promoted their positive thoughts to others and better physical activities (79.7%), established their self-esteem (87.1%), enhanced their social awareness (79.7%), and supported their personal identity (71.4%). The Index showed the mean of 24.39 (*n* = 68) with the range between 12 and 30.

**Table 1:** The results of descriptive analysis among three index categories.

Items	M (SD)	Strongly disagree	Disagree	Undecided	Agree	Strongly agree
Self-developing index	24.39 (3.65)					
My involvement in “intergeneration activities”						
…establish my confidence (*n* = 70)	4.28 (0.72)	0% (0)	1.4% (1)	11.4 (8)	47.1% (33)	40.0% (28)
…support my thoughts and physical activities (*n* = 69)	4.09 (0.81)	0% (0)	4.3% (3)	15.9 (11)	47.8% (33)	31.9% (22)
…enhance my self-esteem (*n* = 70)	3.92 (0.76)	0% (0)	2.9% (2)	25.7% (18)	50.0% (35)	21.4% (15)
…enhance my social awareness (*n* = 69)	4.09 (0.81)	0% (0)	4.3% (3)	15.9 (11)	49.3% (34)	30.4% (21)
…enhance my personal identity (*n* = 70)	3.98 (0.78)	0% (0)	2.9% (2)	25.7 (18)	44.3% (31)	27.1% (19)
Self-Health Index	25.93 (2.96)					
My involvement in “intergeneration activities”						
…positive impact to my life (*n* = 70)	4.43 (0.73)	0% (0)	1.4% (1)	10.0% (7)	34.3% (24)	54.3% (38)
…make me more relax (*n* = 69)	4.49 (0.63)	0% (0)	0% (0)	5.8% (4)	39.1% (27)	55.1% (38)
…relieve my stress (*n* = 70)	4.46 (0.61)	0% (0)	0% (0)	5.7% (4)	44.3% (31)	50.0% (35)
…manage my emotional well-being (*n* = 68)	4.26 (0.65)	0% (0)	0% (0)	11.8% (8)	52.9% (36)	35.3% (24)
…make me stay healthier (*n* = 69)	4.03 (0.76)	0% (0)	2.9% (2)	18.8% (13)	52.2% (36)	26.1% (18)
…enhance my overall well-being (*n* = 68)	4.29 (0.69)	0% (0)	1.5% (1)	8.8% (6)	48.5% (33)	41.2% (28)
Community Building Index	17.73 (1.29)					
My involvement in “intergeneration activities”						
…support my learning of other people (*n* = 67)	4.48 (0.59)	0% (0)	0% (0)	4.5% (3)	43.3% (29)	52.2% (35)
…enhance connection with the community (*n* = 68)	4.21 (0.74)	0% (0)	1.5% (1)	14.7% (10)	45.6% (31)	38.2% (26)
…strengthen my connection to the community (*n* = 69)	4.32 (0.68)	0% (0)	1.4% (1)	7.2% (5)	49.3% (34)	42.0% (29)
…make me understand other people (*n* = 67)	4.64 (0.54)	0% (0)	0% (0)	3.0% (2)	29.9% (20)	67.2% (45)

Regarding the Self-Health Index (SHI), the majority of participants reported positive outcomes from their participation in intergenerational activities. Specifically, 94.3% indicated that the activities improved their quality of life, 94.2% felt more relaxed, 88.6% experienced stress relief, 88.2% noted a positive impact on their emotional well-being, 78.3% believed it supported a healthier lifestyle, and 89.7% felt that their overall well-being was enhanced. The mean score for the SHI was 25.93 (n = 67), falling within the range of 20–30.

In relation to the Community Builders Index (CBI), most participants felt that their involvement in the intergenerational activities helped them better understand others socially (83.8%), fostered stronger connections to their communities (95.5%), contributed to positive community relationships (97.1%), and increased their understanding of diversity (91.3%).

The mean score for the CBI was approximately 17.73 (*n* = 65), with scores ranging from 13 to 20. Bivariate analyses, including one-way ANOVA and correlation coefficients, revealed no statistically significant relationships between (1) participants’ age and their scores on the three Index categories, (2) the duration of time spent at the intergenerational events and the scores on the three Indexes, or (3) the type of contact and the scores on the three Indexes.

Research Question (RQ2): How do older adults perceive their involvement in intergenerational programs as contributing to their health, self-development, and community building?

### Health index (HI)

Regarding health, many participants reported feeling a noticeable improvement in their overall well-being. They highlighted how the activities contributed not only to physical relaxation and stress relief but also positively affected their emotional health. In terms of self-development, participants emphasized the program’s role in enhancing their personal growth, particularly through learning new skills, engaging in cognitive activities, and developing a healthier lifestyle. Finally, with respect to community building, the intergenerational activities were seen as fostering stronger social connections. Participants appreciated how the programs helped them bond with others, promote mutual understanding, and foster positive relationships, contributing to a more cohesive community. The following quotes indicated the responses from the participants.

*“The activities helped me to feel more relaxed. I’ve noticed a significant reduction in my stress levels since joining.” (P1)*

*“Before participating in these events, I often felt tense, but now I feel much calmer and more at ease.” (P2)*

*“I used to have trouble sleeping, but these activities have helped me manage my stress, and now I sleep better.” (P3)*

*“My emotional health has greatly improved. I feel happier and more content since I started taking part in these activities.” (P4)*

*“Being involved in these programs has helped me develop a routine that supports my health, and I feel more energetic.” (P5)*

*“It has been amazing to see how these programs have helped me relax and feel at peace with myself.” (P6)*

*“I’ve noticed that my overall well-being has improved. I feel more positive, and my body feels less stiff after the sessions.” (P7)*

In the context of health, older adults particularly highlighted the stress relief and relaxation they experienced as a result of their involvement in intergenerational programs. Many mentioned that the activities provided them with a sense of calmness and contributed significantly to their emotional well-being. Some participants also reported that the activities helped them maintain a healthier lifestyle by encouraging physical and social engagement. These factors combined to create a sense of holistic health improvement, which they attributed directly to their participation in the programs.

### Self-development index (SDI)

With respect to self-development, participants noted that the intergenerational activities offered them opportunities to engage in new learning experiences, which enriched their personal growth. Many shared how the programs encouraged them to adopt healthier habits, both physically and mentally, leading to better self-management and enhanced self-esteem. In terms of community building, older adults emphasized the programs’ role in fostering stronger connections within their communities. They reported increased understanding of others, reduced social isolation, and the creation of more inclusive environments. These social bonds were seen as critical to their overall sense of belonging and community support.

Self-development:

*“The intergenerational activities have helped me learn new things, such as how to use technology better and stay engaged with the younger generation.” (P1)*

*“I never thought I could learn something new at my age, but these programs have made me feel like I’m growing every day.” (P2)*

*“I’ve learned how to maintain a healthier lifestyle. The advice shared by younger participants on fitness and diet was really helpful.” (P3)*

*“Through these activities, I’ve rediscovered a passion for arts and crafts that I had as a young person. It’s been a great source of joy and personal growth.” (P4)*

*“I feel like I’m more in control of my life now. These programs have taught me how to stay positive and continue developing my skills.” (P5)*

*“The mental stimulation through these programs has kept my mind sharp, and I am constantly learning new things that help me grow.” (P6)*

*“These programs have really helped me build my self-confidence. I’ve been challenged to try new things and embrace change.” (P7)*

### Community building index (CBI)

Older adults generally perceive their involvement in intergenerational programs as significantly contributing to community building in several meaningful ways. Many participants reported that these programs facilitated a stronger sense of social understanding, helping them appreciate the perspectives and experiences of others. Approximately 83.8% of the participants indicated that their participation allowed them to gain a deeper understanding of social dynamics and foster empathy for diverse groups within the community. This aspect of the program not only promoted individual growth but also strengthened interpersonal connections.

Additionally, the majority of participants (95.5%) felt that the intergenerational activities enabled them to connect more deeply with their communities. By engaging in shared experiences across generations, older adults found opportunities to build lasting relationships, enhancing their social network and creating a stronger sense of belonging. Furthermore, 97.1% of the participants noted that their involvement helped establish positive community relations, emphasizing the role of these programs in bridging generational gaps and promoting social cohesion. This sense of mutual respect and understanding also extended to recognizing and celebrating differences, with 91.3% of participants reporting an improved ability to appreciate diversity within their communities.

*“I feel much more connected to my community now. I’ve met so many wonderful people through these activities.” (P1)*

*“I’ve gained a better understanding of others. The younger generations teach me new things, and I’ve learned to appreciate our differences.” (P2)*

*“Before I joined, I felt isolated, but now I’ve made so many new friends. I truly feel part of the community.” (P3)*

*“The sense of unity and shared purpose we have in these programs is incredible. It feels like a big family.” (P4)*

*“These activities helped me understand the challenges others face, and I believe that makes me more compassionate and empathetic.” (P5)*

*“I’ve learned so much about my community through these programs. It has helped me feel like I’m part of something bigger.” (P6)*

*“I used to feel disconnected, but now I feel more involved. These programs have bridged the gap between different generations and brought us closer.” (P7)*

Overall, older adults viewed their involvement in intergenerational programs as a vital means of fostering community engagement, both on a personal level and within the broader societal context. The programs offered them opportunities to contribute to and benefit from a sense of collective identity, reinforcing the importance of intergenerational interaction in enhancing community bonds.

## Discussion

The findings of this study reveal that older adults perceive their involvement in intergenerational programs as a significant contributor to community building. Participants consistently expressed that these programs facilitated social understanding, improved community connections, and promoted positive relationships across generations. Approximately 83.8% of participants reported gaining a deeper understanding of others in their communities, highlighting the potential of intergenerational activities to foster empathy and bridge social gaps. This finding is consistent with prior research, which has demonstrated that intergenerational programs can enhance participants’ social awareness and understanding of diverse perspectives (Furuto, 2019). The ability to build these connections contributes to social cohesion, as participants feel more connected to their broader community (Murray et al., 2025; Pinazo-Hernandis and Carrascosa, 2025).

The majority of participants in this study (95.5%) reported that intergenerational activities allowed them to connect more deeply with their communities. This aspect of community engagement aligns with previous studies, which suggest that older adults often feel isolated, but that participation in intergenerational programs provides opportunities to combat this isolation and develop a stronger sense of belonging (Tucker, 2021). The sense of connection observed in this study is also supported by research indicating that such programs facilitate the formation of long-lasting relationships between individuals from different generations, strengthening the social fabric of communities (Rabinowitz et al., 2022). The collaborative nature of these programs, where individuals of all ages contribute and share experiences, fosters a greater sense of unity within the community (Larkin and Pines, 2020).

Furthermore, 97.1% of participants in this study indicated that intergenerational programs contributed to establishing positive community relations. This finding reflects the growing recognition that these programs play a critical role in creating more cohesive, inclusive societies by fostering mutual respect between different generations. According to a study by Saito et al. (2020), positive intergenerational interactions can help reduce stereotypes and misconceptions between young and old, improving relationships across age groups. The ability to create these positive relationships was evident in this study, as older adults saw their participation as a way to directly contribute to the harmony and inclusiveness of their communities (Morioka et al., 2019). This finding underscores the broader social impact of intergenerational initiatives, which not only benefit the participants but also have far-reaching effects on community dynamics.

The ability to recognize and appreciate differences within the community was also highlighted by 91.3% of the participants in this study, who felt more attuned to diversity through their involvement in the program. This aspect of intergenerational programs is essential for fostering inclusivity and acceptance. Previous studies have found that such programs can increase participants’ awareness of cultural and generational differences, enhancing social integration (Johnson et al., 2018; Nakagawa and Okamoto, 2021). The present study corroborates these findings, showing that older adults involved in intergenerational activities develop a greater appreciation for diversity, which in turn enhances their sense of belonging and engagement within the community. The positive influence on community building through intergenerational understanding is evident across a range of studies and is a key strength of these programs (Kuehne et al., 2017).

In comparison to similar studies, the findings of this research both align with and expand on existing literature. While the core outcomes—such as fostering social understanding, improving community connections, and establishing positive relationships—are consistent with earlier studies (Saito et al., 2020; Tucker, 2021; Suryanti et al., 2022; Yechenming et al., 2026), this study highlights the particularly strong sense of community contribution reported by older adults. Unlike some studies that focus primarily on the benefits of intergenerational programs for younger participants or program facilitators, this study provides insight into how older adults themselves perceive their roles within these programs, positioning them not just as recipients of benefits but as active contributors to community building (Murray et al., 2025; Pinazo-Hernandis and Carrascosa, 2025; Rabinowitz et al., 2022; He et al., 2024). This nuanced perspective adds depth to the literature and emphasizes the value of viewing older adults as integral members of the community who actively contribute to social development.

However, these findings should be interpreted in light of important regional limitations. The study was conducted within a specific socio-cultural context characterized by strong collectivist traditions, established neighborhood networks, and locally supported community organizations, which may have shaped older adults’ perceptions of contribution and social responsibility. In regions where community infrastructure is less developed, where intergenerational contact is more fragmented, or where individualistic cultural norms predominate, older adults may not experience or articulate the same degree of agency and community engagement. Additionally, variations in economic development, access to public services, and local policy support for active aging initiatives could significantly influence program outcomes. Therefore, while the present findings enrich the broader literature on intergenerational programs, their transferability to other geographic, socio-economic, or cultural contexts should be approached with caution. Future comparative studies across diverse regional settings are needed to examine how structural, cultural, and policy environments mediate the relationship between intergenerational participation and perceived community contribution.

## Conclusion

The findings of this study underscore the significant role that intergenerational programs play in enhancing community building among older adults, fostering social understanding, community connections, and positive relationships across generations. These programs not only promote individual well-being but also contribute to a more inclusive and cohesive community. The implications of these findings suggest that community organizations and policymakers should continue to invest in and promote intergenerational initiatives as a means of fostering social integration and reducing isolation among older adults. However, the study’s limitations include its reliance on self-reported data, which may be subject to biases, and the focus on a specific geographic area, limiting the generalizability of the findings. Future research could explore the long-term effects of such programs across diverse populations and settings, incorporating both qualitative and quantitative measures to further validate the impact of intergenerational programs on community development and individual well-being.

Beyond its empirical contributions, this study advances several theoretical and contextual innovations grounded in the socio-cultural setting of Sichuan Province. First, it extends social capital and intergenerational solidarity frameworks by incorporating the concept of culturally embedded reciprocity shaped by Confucian filial norms and the collectivist community structure prevalent in Sichuan. Unlike Western individual-centered aging models, the mechanism observed in this study reflects a relational governance logic in which older adults’ social participation is mediated through neighborhood committees, community cultural halls, and family-based moral expectations. The analysis further identifies a localized mechanism of “emotion-driven cohesion,” whereby shared regional identity, dialect, and cultural practices (e.g., community square dancing and traditional festivals) function as affective bridges between generations. This contextual interpretation suggests that intergenerational programs in Sichuan operate not merely as structured activities but as culturally resonant platforms that activate latent social trust and mutual obligation. Theoretically, this refines existing community-building models by demonstrating how macro-level cultural values and meso-level community institutions interact to shape micro-level psychosocial outcomes among older adults. Such insights highlight the importance of embedding intergenerational interventions within local socio-cultural ecosystems rather than adopting decontextualized program models.

## Data Availability

The raw data supporting the conclusions of this article will be made available by the authors, without undue reservation.
